# Standardized Opioid Prescription Protocol Reduces Opioid Consumption After Total Joint Arthroplasty

**DOI:** 10.5435/JAAOSGlobal-D-19-00163

**Published:** 2019-12-11

**Authors:** Kenneth M. Vaz, Philip S. Huang, Steven N. Copp

**Affiliations:** From the Department of Orthopaedic Surgery, Scripps Clinic, La Jolla, CA.

## Abstract

Previous studies demonstrate routine overprescription of pain medications after total joint arthroplasty (TJA). A standardized reduced opioid prescription protocol for TJA was initiated at our institution, which we hypothesized would lead to a reduction in opioid consumption compared with patients who had surgery before initiation of the new protocol.

**Methods:**

We evaluated 97 consecutive opioid-naive patients undergoing primary total knee arthroplasty (TKA) and total hip arthroplasty (THA) using a standardized opioid prescription protocol (standardized group). A control subject group consisted of 99 patients undergoing TKA and THA just before the adaptation of the standardized prescribing protocol (historic group). Postoperatively, patients brought their remaining pain medication to their 1-month follow-up visit. The number of pills consumed was counted and converted into oral morphine equivalents (OME). Current pain level and the need for pain medication refill was assessed.

**Results:**

Among TKA patients, mean opioid consumption in the standardized group (48.5 pills; 432 OME) was markedly less than the historic group (76.2 pills; 903 OME) (both *P* < 0.01). Refills were required in 50% of the standardized group and 29% of the historic group (*P* = 0.038). Average pain scores for the standardized and historic groups were 2.3 and 3.2, respectively (*P* = 0.057). Among THA patients, mean opioid consumption in the standardized group (19.1 pills; 200 OME) was markedly less than the historic group (41.3 pills; 504 OME) (both *P* < 0.01). Refills were required in 16% of the standardized group and 8% of the historic group (*P* = 0.263). Average pain scores for the standardized and historic groups were 1.7 and 1.8, respectively (*P* = 0.608).

**Discussion:**

Initiation of a standardized opioid prescribing protocol after TJA for opioid-naive patients led to a reduction in opioid consumption, but resulted in an increased need for refills.

The opioid epidemic in the United States has highlighted the importance of appropriate prescribing practices after total joint arthroplasty (TJA), especially considering the increasing frequency with which the procedure is done in the United States.^1–3^ Given that many patients who abuse opioids start with medications that are left over from previous prescriptions, prescribing the appropriate amount of narcotics postoperatively is critical in minimizing the risk of abuse and overdose.

The use of multimodal analgesia and rapid recovery protocols have allowed patients to further minimize opioid exposure and their side effects including nausea, pruritis, constipation, somnolence, delirium, and dependence, all of which can inhibit recovery.^4,5^ The use of acetaminophen, NSAIDs, oral steroids, and anticonvulsants (gabapentin and pregabalin) has been useful adjuncts in pain management to reduce the need for opioids for pain control.^4,6–8^

Although there are data demonstrating that multimodal pain management has been effective in reducing opioid consumption in the hospital, there are relatively little data determining the average opioid consumption in the postdischarge period with the above.^4^ These data become more important as more patients are being discharged to home less than 24 hours after surgery and are reliant on their postoperative prescriptions for appropriate pain control.^9^ The American Academy of Orthopaedic Surgeons recommended a standardized protocol for opioid prescription after TJA, but no recommendations were made regarding quantities of pills or oral morphine equivalents (OME).^10^

Our group has previously reported on average narcotic consumption after routine TJA. We found that narcotic pain medications were overprescribed after both total knee arthroplasty (TKA) and total hip arthroplasty (THA) by 34% and 140%, respectively.^11^ Given those findings, we have prescribed narcotic pain medication at a reduced and standardized quantity postoperatively.

Although previous studies evaluating reduced opioid protocols have demonstrated success in reducing OME prescribed to patients, no study to date has determined whether reduced standardized prescriptions lead to less opioid consumption or the amount of pills remaining in the patient's possession in the early postoperative period.

The purpose of this observational follow-up study was to determine whether a standardized protocol for opioid prescriptions reduced pills remaining and opioid consumption. We hypothesized that patients would consume less OME and have less pills remaining with a standardized prescribing pattern.

## Methods

A total of 97 patients undergoing primary THA and TKA from a single surgeon from July 2018 to January 2019 were prospectively selected to receive a standardized postoperative opioid prescription and compared with a historical cohort of 99 patients who underwent THA and TKA from July 2017 to January 2018. We included patients treated by a single surgeon to control for variability in surgical technique and perioperative pain management regimens. Patients were excluded if they underwent a revision surgery, had bilateral procedures, deviated from standard inpatient pain protocol outlined below, had incomplete data, or were discharged to a skilled nursing facility. Patients were also excluded if they had a history of opiate dependency or narcotic abuse, which was verified through the use of a government registry (CURES California). We defined opioid dependency as use greater than 3 months before the patient's surgical procedure, which has been used as criteria in previous opioid-related research.^12^

In all patients, TKA was done through a standard medial parapatellar approach and THA through a standard posterolateral approach. Patients were mobilized on postoperative day 0 and were discharged once pain was adequately controlled, and they met physical therapy criteria. The mean length of stay was 1.3 days (range, 1 to 4 days). All patients in both cohorts received a standard inpatient postoperative pain protocol including intravenous opioids for breakthrough pain in addition to oral narcotic pain medications. Patients received 5 or 10 mg of oxycodone for moderate or severe pain, respectively, with scheduled acetaminophen. Patients received celecoxib 200 mg twice daily while in the hospital. A short course of oral steroids was prescribed, in the form of dexamethasone 4 mg taper while in the hospital.

Patients in the historic prescribing practice cohort were given a postoperative pain medication prescription at the inpatient provider's discretion. THA and TKA in the standardized prescription cohort were given 30 and 40 pills, respectively, with dose dependent on the patient's pain medication requirements while in the hospital.

Patient characteristics (age, sex, body mass index, and type of surgery) and narcotic prescription information (type of narcotic prescribed, dosage, and number of pills) were extracted from medical records and verified with the use of a government database (CURES California). All patients were called before their 1-month follow-up visit and asked to bring their remaining prescription pain medication to their appointment. At the 1-month follow-up visit, the number of pain pills remaining in each patient's prescription bottle was manually counted and recorded. To account for the differences in types of opioids consumed, dosages were converted into OME using a common conversion formula.^13^ Pain scores were assessed using the Numeric Rating Scale. Refills of pain medication were retrieved through a government database and verified. The average number of refills per patient was defined as the total number of refills requested within the cohort (as some patients requested a refill more than once) divided by the total number of patients.

Descriptive statistics were used to summarize patient characteristics. Frequencies and ranges were used to summarize categorical variables, and mean values and SDs were calculated to summarize continuous variables. Differences in opioid consumption between historic and standardized prescribing practices were compared with regard to total number of pills prescribed, pills remaining, OME consumed, and average refills per patient. Statistical significance was set at α < 0.05. All tests were two-tailed.

## Results

Ninety-seven patients (51 THA and 46 TKA) met the inclusion criteria and were included in the study. Patient demographics between the standardized prescription and historic group were comparable with regard to sex, age, body mass index, and duration of follow-up (Table 1).

**Table 1:** Demographics for Historic and Standardized Protocol Cohorts

THA	Historic Cohort (n = 48)	Standardized Cohort (n = 51)	*P* Value
Age	66.0	67.4	0.49
Male (%)	54	39	0.14
BMI	27.8	26.7	0.28
Follow-up, days	30.2	30.2	0.95

TKA	Historic Cohort (n = 51)	Standardized Cohort (n = 46)	*P* Value
Age	68.0	69.5	0.41
Male (%)	41	35	0.52
BMI	28.4	29.5	0.32
Follow-up, days	28.8	30.0	0.15

BMI = body mass index, THA = total hip arthroplasty, TKA = total knee arthroplasty

Findings for both cohorts are summarized in Table 2. When compared with previous practice, patients in the standardized prescribing practice group had less pills prescribed for TKA (115 versus 62, *P* < 0.001) and THA (94 versus 35, *P* < 0.001). Patients had less pills remaining at their first follow-up for both TKA (39 versus 15, *P* < 0.001) and THA (53 versus 16, *P* < 0.001). Patients consumed significantly less pill quantities after TKA (76 versus 49, *P* < 0.01) and THA (41 versus 19, *P* < 0.001) with a standardized protocol along with less OME consumed in both TKA (902 versus 432, *P* < 0.001) and THA (504 versus 200, *P* < 0.001).

**Table 2:** Postoperative Narcotic Consumption for All Patients

THA	Historic Cohort (n = 48)	Standardized Cohort (n = 51)	*P* Value
Pills prescribed	94	35	<0.001
Pills remaining	53	16	<0.001
OME consumed	504	200	<0.001
Refills/patient	0.1	0.2	0.26
NRS pain score	1.8	1.7	0.61

TKA	Historic Cohort (n = 51)	Standardized Cohort (n = 46)	*P* Value
Pills prescribed	115	62	<0.001
Pills remaining	39	15	<0.001
OME consumed	902	432	<0.001
Refills/patient	0.3	0.7	0.02
NRS pain score	3.2	2.3	0.06

NRS = Numeric Rating Scale, OME = oral morphine equivalents, THA = total hip arthroplasty, TKA = total knee arthroplasty

When comparing patients in each cohort who did not request a refill, OME consumed was also decreased in both TKA (649 versus 265, *P* = 0.002) and THA (440 versus 160, *P* = 0.004) with a standardized protocol. The total number of refills per patient within the first month ranged from one to four times for TKA and one to three times for THA. The refill rate was higher in the standardized prescribing cohort with both TKA (29% versus 50%, *P* = 0.04) and THA (8% versus 16%, *P* = 0.26). There were more average refills per patient in the standardized prescribing group with both TKA (0.7 versus 0.3, *P* = 0.02) and THA (0.2 versus 0.1, *P* = 0.26).

Pain scores were lower with standardized prescribing practices versus historic for both THA (1.8 versus 1.7, *P* = 0.61) and TKA (3.2 versus 2.3, *P* = 0.06) cohorts, although in both cases, differences were less than the minimum clinically important difference of that has been reported, and not statistically significant.^14^

## Discussion

A previous study has suggested that similar to spine and upper extremity surgery, opioids are typically overprescribed for TJA, but there have been little data to suggest what an appropriate amount may be.^13^ Other previous studies have evaluated the number of prescriptions given to patients and the associated OME prescribed, but no study to the best of our knowledge has evaluated the actual amount of opioids consumed in the postdischarge period after TJA when a standardized protocol was implemented.^15,16^ This is the first study to demonstrate that with a reduced opioid prescription, the quantity of pills and OME consumed is also reduced after TJA.

Although it would be reasonable to expect that the OME consumed may be unchanged between cohorts with a reduced number of pills remaining. We found that the OME consumption also decreased by greater than 50% in both THA and TKA when standardizing the quantity of the initial postoperative prescription.

This was not only found to apply to all patients but also in patients who did not request a refill. Patients who did not request a refill still consumed less OME with a standardized prescription. In the case of THA, this averaged to 37 fewer oxycodone 5 mg pills and in the case of TKA, 63 fewer oxycodone 5 mg pills in the first month when compared with our traditional prescribing practice.

There have been concerns that with reduced amounts of opioids that patient perceived pain would be increased, although this does not seem to be the case with pain scores being comparable between both THA and TKA cohorts.

Previous studies have demonstrated that refill rates were not markedly changed when restricting postoperative opioid prescriptions, but we found an increase in the average number of refills per patient.^15,16^ Increased refills may also decrease opioid consumption. Patients who need a refill are typically advised to reduce their dosing or given lower strength prescriptions when compared with their initial postoperative prescription. They are also encouraged to use non-narcotic measures for pain control. Although there may be some benefit from the perspective of narcotic consumption when restricting postoperative pill quantities, this does have to be weighed against the time of office staff and providers to provide refills and counsel patients. An average refill rate per patient has not been reported previously and may be a useful metric in determining the extra resources that need to be dedicated to pursuing a standardized opioid protocol. Assuming a minimum of 10 minutes of staff time per refill to include counseling a patient, checking the government registry for abuse which is now required by law, and contacting a provider to approve a refill, at least an extra 5 minutes of staff and provider time for every TKA and 1 minute of time for every THA would need to be accounted for with our protocol.

The strengths of this study include strict exclusion criteria and use of a sample of patients treated by a single surgeon to control for confounding variables with similar demographics. We physically counted the number of pills remaining for each patient as opposed to determining the number of prescriptions administered as has been done in previous studies to determine true consumption. We were also able to account for patients who requested multiple refills because this also has a magnified influence on provider time and resources when contrasted with determining percentage of patients who request a refill. We converted all medications to OME to account for different types of opioids prescribed. We believe these methods ensured that we were able to effectively capture all oral narcotics consumed. The use of a government registry also minimized concerns regarding recall bias or chart documentation as any narcotic prescriptions that were filled in the state by any provider postoperatively were accounted for. Our findings of the average OME consumption at 1 month are consistent with a previously cited study where the maximum prescription was 400 OME after TJA.^15^ We also found that this threshold may be reduced in the setting of THA.

There are some weaknesses associated with our study. Our sample size from a single institution is limited. For patients who received refills, we were unable to standardize what medications they had refilled. Given the nature of how patients experience pain, there was wide variation in how many pills patients consumed even in the setting of a standardized protocol. Given the wide variation in the number of pills consumed, future work will focus both on which group of patients it may be possible to further reduce or forego narcotic pain medication altogether and which groups in the opioid-naive patient cohort undergoing TJA may be at risk of requiring high doses of narcotics or have the potential for dependence.
